# Robot-Assisted Laparoscopic Repair of Spontaneous Appendicovesical Fistula

**DOI:** 10.1089/cren.2016.0063

**Published:** 2016-06-01

**Authors:** Yusuf Kibar, Serdar Yalcin, Burak Kopru, Engin Kaya, Bahadir Topuz, Turgay Ebiloglu

**Affiliations:** ^1^ Department of Urology, Gulhane Military Medical Academy, Ankara, Turkey.; ^2^ Department of Urology, Agrı Military Hospital, Agrı, Turkey.; ^3^ Department of Urology, Konya Military Hospital, Konya, Turkey.; ^4^ Department of Urology, Etimesgut Military Hospital, Ankara, Turkey.

## Abstract

***Background:*** To report the first case of the spontaneous appendicovesical fistulas' (AVF) repair with robot assisted laparoscopy.

***Case Presentation:*** A 29-year-old male patient with urgent persistant bacteriuria and dysuria was referred to our clinic. Physical examination and blood tests were normal. He had used various antibiotics due to recurrent UTI for about 20 years. Computed tomography revealed the fistula tract between the distal end of the appendix and right lateral wall of the bladder dome. He was successfully treated with robot-assisted laparoscopic repair. Following this surgery, the patient's complaints were resolved completely.

***Conclusion:*** AVF is the rare condition. Robot-assisted laparoscopy repair of AVF is safe and effective treatment option.

## Introduction and Background

Appendicovesical fistula (AVF) is a chronic disease that can develop between appendix and bladder because of an inflammatory process.^1^ AVF is a rare cause for recurrent urinary tract infection (UTI).^2^ AVF may occur because of benign or malignant diseases as it may also occur spontaneously. The diagnosis may be very confusing because of nonspecific symptoms. After diagnosis, it can be treated by open or laparoscopic surgery. According to the literature, AVF was often treated with open surgery and in a limited number of cases laparoscopic method was also practiced. To the best of our knowledge we performed the first robot-assisted laparoscopic repair of AVF in a spontaneous AVF case. Our primary goal is to explain the technique of robot-assisted laparoscopic approach in a spontaneously occurred AVF case for the patient with recurrent UTI.

## Presentation of Case

A 29-year-old male patient was admitted to our clinic with persistent bacteriuria, dysuria, and urgency. In the patient's history, it was found that he had used various antibiotics because of recurrent urinary tract infection (UTI) for about 20 years. He had pneumaturia episodes time to time. There was no specific indication on physical examination. There was no abnormality in blood count or biochemical examination too. In urinalysis, pyuria and nitrite positivity were found. Escherichia coli and Enterobacteriaceae growth were detected in the urine culture. According to the antibiogram, the patient was treated with intravenous ciprofloxacin 400 mg/day.

**FIG. 1.**
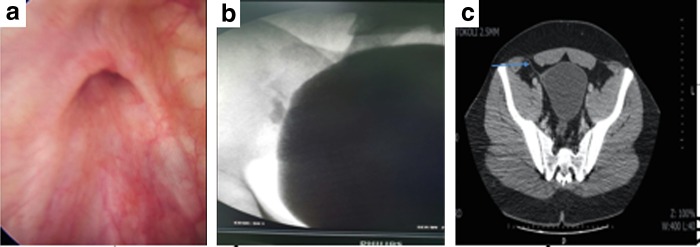
**(a)** Cystoscopic image of appendicovesical fistula (AVF) in right lateral wall of the bladder dome. **(b)** Contrast agent near the right side of the bladder in voiding cystography. **(c)** Detected a fistula tract between the distal end of the appendix and right lateral wall of the bladder dome in CT scan.

Because of recurrent UTI, cystoscopy was performed and nearly 1 cm diameter fistula opening was observed on the right lateral wall of the bladder dome (Fig. 1a).

Contrast agent crossing through the fistula tract was seen in voiding cystography (Fig. 1b).

A fistula tract between the distal end of the appendix and right lateral wall of the bladder dome was detected in the CT. Free air images in bladder and increased bladder wall thickness were also detected (Fig. 1c).

**FIG. 2.**
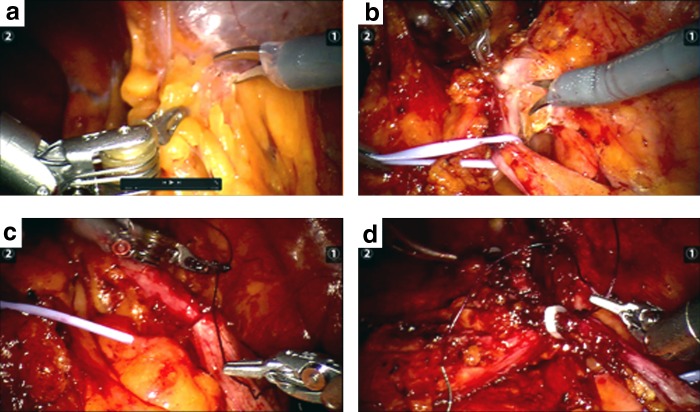
**(a–d)** The images of robot-assisted laparoscopic AVF repair.

Robot-assisted laparoscopic AVF repair was performed. A three-port configuration was used with an additional 10 mm assistant port. After providing the pneumoperitoneum with the help of Veress needle, a 12 mm trocar was placed just 2 cm above the umbilicus as a camera port. Two of the 8 mm robot trocars were placed to the lateral edges of rectus abdominis muscle, targeting appendix and right lateral wall of the bladder. A 10 mm assistant trocar was placed between the camera and left robotic trocar. After docking the robot, cecum and appendix were found in a mass attached to the anterior wall of the abdomen (Fig. 2a).

After dissecting this attached mass, appendix, right lateral wall of the bladder, and about 2 cm fistula tract were revealed (Fig. 2b).

The appendix was mobilized, and 2-0 Vicryl (Ethicon, Inc., Somerville, NJ) and 2-0 Monocryl (Ethicon, Inc.) sutures were placed at the base of the appendix and the appendix was separated from the cecum (Fig. 2c).

Bladder side of the fistula was ligated with 10 mm Hem-O-Lok clips (Teleflex Medical, Research Triangle Park, NC) and sutured with 2-0 Vicryl (Fig. 2d). The fistula tract was excised and removed from the assistant trocar.

Water tightness of bladder was checked with saline and no leakage was detected. Silicone drain and urethral catheter were left in the patient. The drain and urethral catheter were removed on third and seventh days after surgery, respectively. The patient was discharged without complications at the seventh day.

After the urethral catheter had been removed, his recurrent cystitis episodes disappeared.

## Discussion and Literature Review

AVF is a very rare condition and accounts for less than 5% of whole vesicoenteric fistulas.^3^ Although it can be observed in both children and adults, it is more common in males between 10 and 40 years.^2^ The reason for less frequent AVF cases in females is that the uterus serves as a barrier place between bladder and appendix.^3^ Our male patient was also in this same age group.

The most common cause of AVF is acute appendicitis^2^ and the others can be listed as adenocarcinoma, carcinoid tumor, neuroma, Crohn's disease, villous adenoma, actinomyces, Meckel's diverticulum, and Hirschsprung's disease.^3–5^ Although AVF has occurred spontaneously without any disease and comorbidities in our case, it can be revealed after appendicitis.

The patients with AVF have commonly frequent dysuria, abdominal pain, fever, and chills, similar to UTI complaints.^6^ More specific symptoms can be pneumaturia and fecaluria.^6^ *Escherichia coli* and Klebsiella are detected more common in urine cultures.^2^ A history of recurrent UTI and repeated antibiotic use, pneumaturia, and Enterobacteriaceae and Escherichia coli growth in urine cultures were detected in our case.

Diagnosis of AVF is difficult without imaging methods at the beginning of disease. Some of the imaging methods can be used to show the fistula tract. Although ultrasonography does not have any benefit for diagnosis, voiding cystography, barium enema, CT, and magnetic resonance imaging can be used for diagnosis instead.^7^ Voiding cystography could be useful for showing fistula tract between bladder and appendix.^3^ Calcified fecaloma on bladder wall, air bubble in bladder cavity, and fistula tract can be detected with CT.^8^ Colonoscopy and cystoscopy should be performed on patients when considering AVF. Colonoscopy is a useful tool to exclude possible intestinal pathologies. Edema and opening of fistula in the bladder wall can be seen with cystoscopy.^3^ In our case, voiding cystography, CT, and cystoscopy were performed. Opening of fistula was located at the right lateral wall of bladder dome and the fistula tract between bladder and appendix was determined using these methods.

The first option in the treatment of AVF is to use appropriate antibiotics. Surgical procedures are appendectomy, excision of fistula tract, and bladder wall repair with/without partial cystectomy.^2^ Open or laparoscopic surgical approaches can be used for treatment. Although laparotomy has been used for diagnosis and treatment for many years, recently laparoscopic treatment is used for appropriate patients.^9^ Albrecht et al. reported their two cases of laparosopic experience on AVF. Crohn's disease and chronic appendicitis complication were reported as the etiology of these cases in their study. Both of these cases were repaired by laparoscopic appendectomy.^9^ Also Chung et al. reported laparoscopic repair of AVF. In that case, laparoscopic repair was performed transabdominally through three ports. The fistula was excised by an Endo-GIA stapler while binding appendix with a 2-0 Vicryl. During excision, flexible cystoscopy was performed to monitor the bladder inside.^2^ Lee et al. reported an AVF case due to Meckel diverticulum and laparoscopic repair of AVF. In that case, they also used three laparoscopic ports for the procedure. Appendectomy, fistula tract excision, and partial cystectomy were performed. Ten millimeter Hem-O-Lok clip and 2-0 Vicryl were used for appendectomy. Bladder was repaired in a watertight two-layer manner with 3-0 Vicryl.^3^ Another AVF case was reported by Garcia-Munos-Najar et al. and they determined the fistula tract using CT. They treated their case using laparoscopic approach.^9^

In our case, we performed robot-assisted laparoscopic repair of spontaneous AVF using a three-port configuration with an extra 10 mm assistant port as already described. After appendectomy and the fistula tract removal, we have repaired the bladder wall without partial cystectomy and cecum using sutures and weck clip. Surgery lasted ∼45 minutes. Intraoperative bleeding was insignificant. Acetaminophen and metamizol were used for postoperative pain management. Narcotic analgesic like morphine was not needed.

## Conclusion

More than 100 AVF repair cases treated with open and laparoscopic surgical approaches are reported in the literature. But there is no report for robot-assisted laparoscopic AVF repair. To the best of our knowledge, this is the first case of AVF repair in the world by using da Vinci surgical system. In recent years, taking into account the progress in minimally invasive surgery in urology, robotic surgery stands out. It is possible to add AVF repair to increase the application area of robotic surgery. AVF repair using robotic surgery is an effective and safe technique for appropriate patients.

## Abbreviations Used

AVF: appendicovesical fistula
CT: computed tomography
UTI: urinary tract infection
